# Supplementary material for: Modulation of Transcription Profile Induced by Antiproliferative Thiosemicarbazone Metal Complexes in U937 Cancer Cells
Source: Pharmaceutics. 2023 Apr 24;15(5):1325. doi: 10.3390/pharmaceutics15051325 (PMC10220753; doi:10.3390/pharmaceutics15051325)
Supplement: Supplementary file 1 [file pharmaceutics-15-01325-s001.zip › pharmaceutics-2291386-supplementary.pdf]

Table S1. Effects of [Ni(tcitr)$_2$], [Pt(tcitr)$_2$] and [Cu(tcitr)$_2$] on the expression of RRM1. U937 cells were seeded (2 × 10^6 cells/flask) into 25 cm^2 flasks with complete medium and then treated with GI_{50} value for each metal complexes for 1 – 4-24 h. Total RNA was extracted, quantified and 1 μg was reverse-transcribed. The complementary DNA (cDNA) was used as a template of qRT-PCR reactions. Data are expressed as fold change ± standard deviation in target gene expression in treated cells normalized to the internal control gene (GAPDH) and relative to the DMSO negative control. Fold change values >2 mean an up regulation of the target gene; fold change values < 0.5 mean a down regulation of the target gene. Basal expression was defined by a fold change ranging from 0.5 to 1.9

RRM1	[Ni(tcitr)$_2$]	[Pt(tcitr)$_2$]	[Cu(tcitr)$_2$]
**1h**	1.48 ± 0.02	0.61 ± 0.18	1.49 ± 0.09
**4h**	0.65 ± 0.04	0.37 ± 0.05	1.61 ± 0.03
**24h**	1.16 ± 0.39	0.42 ± 0.04	0.68 ± 0.09

Table S2. Effects of [Ni(tcitr)$_2$], [Pt(tcitr)$_2$] and [Cu(tcitr)$_2$] on the expression of RRM2. U937 cells were seeded (2 × 10^6 cells/flask) into 25 cm^2 flasks with complete medium and then treated with GI_{50} value for each metal complexes for 1 – 4-24 h. Total RNA was extracted, quantified and 1 μg was reverse-transcribed. The complementary DNA (cDNA) was used as a template of qRT-PCR reactions. Data are expressed as fold change ± standard deviation in target gene expression in treated cells normalized to the internal control gene (GAPDH) and relative to the DMSO negative control. Fold change values >2 mean an up regulation of the target gene; fold change values < 0.5 mean a down regulation of the target gene. Basal expression was defined by a fold change ranging from 0.5 to 1.9

RRM2	[Ni(tcitr)$_2$]	[Pt(tcitr)$_2$]	[Cu(tcitr)$_2$]
**1h**	27.28 ± 2.45	0.61 ± 0.04	0.69 ± 0.08
**4h**	18.07 ± 3.42	0.40 ± 0.03	0.65 ± 0.01
**24h**	246.64 ± 12.27	0.62 ± 0.04	1.16 ± 0.12

Table S3. Effects of [Ni(tcitr)$_2$], [Pt(tcitr)$_2$] and [Cu(tcitr)$_2$] on the expression of p53R2. U937 cells were seeded (2 × 10^6 cells/flask) into 25 cm^2 flasks with complete medium and then treated with GI_{50} value for each metal complexes for 1 – 4-24 h. Total RNA was extracted, quantified and 1 µg was reverse-transcribed. The complementary DNA (cDNA) was used as a template of qRT-PCR reactions. Data are expressed as fold change ± standard deviation in target gene expression in treated cells normalized to the internal control gene (GAPDH) and relative to the DMSO negative control. Fold change values >2 mean an up regulation of the target gene; fold change values < 0.5 mean a down regulation of the target gene. Basal expression was defined by a fold change ranging from 0.5 to 1.9

p53R2	[Ni(tcitr)$_2$]	[Pt(tcitr)$_2$]	[Cu(tcitr)$_2$]
**1h**	0.48 ± 0.01	0.26 ± 0.00	1.02 ± 0.06
**4h**	0.41 ± 0.02	0.28 ± 0.02	1.09 ± 0.01
**24h**	0.94 ± 0.18	1.56 ± 0.15	1.25 ± 0.18

Table S4. Effects of [Ni(tcitr)$_2$], [Pt(tcitr)$_2$] and [Cu(tcitr)$_2$] on the expression of ATM. U937 cells were seeded (2 × 10^6 cells/flask) into 25 cm^2 flasks with complete medium and then treated with GI_{50} value for each metal complexes for 1 – 4-24 h. Total RNA was extracted, quantified and 1 μg was reverse-transcribed. The complementary DNA (cDNA) was used as a template of qRT-PCR reactions. Data are expressed as fold change ± standard deviation in target gene expression in treated cells normalized to the internal control gene (GAPDH) and relative to the DMSO negative control. Fold change values >2 mean an up regulation of the target gene; fold change values < 0.5 mean a down regulation of the target gene. Basal expression was defined by a fold change ranging from 0.5 to 1.9

ATM	[Ni(tcitr)$_2$]	[Pt(tcitr)$_2$]	[Cu(tcitr)$_2$]
**1h**	0.13 ± 0.01	1.19 ± 0.22	0.64 ± 0.07
**4h**	1.55 ± 0.12	1.12 ± 0.36	0.13 ± 0.04
**24h**	0.83 ± 0.10	1.26 ± 0.23	1.89 ± 0.21

Table S5. Effects of [Ni(tcitr)$_2$], [Pt(tcitr)$_2$] and [Cu(tcitr)$_2$] on the expression of Chk2. U937 cells were seeded (2 × 10^6 cells/flask) into 25 cm^2 flasks with complete medium and then treated with GI_{50} value for each metal complexes for 1 – 4-24 h. Total RNA was extracted, quantified and 1 µg was reverse-transcribed. The complementary DNA (cDNA) was used as a template of qRT-PCR reactions. Data are expressed as fold change ± standard deviation in target gene expression in treated cells normalized to the internal control gene (GAPDH) and relative to the DMSO negative control. Fold change values >2 mean an up regulation of the target gene; fold change values < 0.5 mean a down regulation of the target gene. Basal expression was defined by a fold change ranging from 0.5 to 1.9

Chk2	[Ni(tcitr)$_2$]	[Pt(tcitr)$_2$]	[Cu(tcitr)$_2$]
**1h**	15.62±4.56	0.39±0.25	0.11±0.07
**4h**	0.03±0.00	0.55±0.22	0.06±0.03
**24h**	1.31±0.05	5.5±0.91	0.02±0.00

Table S6. Effects of [Ni(tcitr)$_2$], [Pt(tcitr)$_2$] and [Cu(tcitr)$_2$] on the expression of ATR. U937 cells were seeded (2 × 10^6 cells/flask) into 25 cm^2 flasks with complete medium and then treated with GI_{50} value for each metal complexes for 1 – 4- 24 h. Total RNA was extracted, quantified and 1 μg was reverse-transcribed. The complementary DNA (cDNA) was used as a template of qRT-PCR reactions. Data are expressed as fold change ± standard deviation in target gene expression in treated cells normalized to the internal control gene (GAPDH) and relative to the DMSO negative control. Fold change values >2 mean an up regulation of the target gene; fold change values < 0.5 mean a down regulation of the target gene. Basal expression was defined by a fold change ranging from 0.5 to 1.9

ATR	[Ni(tcitr)$_2$]	[Pt(tcitr)$_2$]	[Cu(tcitr)$_2$]
**1h**	0.20 ± 0.07	0.82 ± 0.07	0.45 ± 0.11
**4h**	1.81 ± 0.32	0.94 ± 0.12	0.04 ± 0.01
**24h**	0.66 ± 0.15	1.45 ± 0.10	1.77 ± 0.15

Table S7. Effects of $[Ni(tcitr)_2]$, $[Pt(tcitr)_2]$ and $[Cu(tcitr)_2]$ on the expression of Chk1. U937 cells were seeded (2 × 10^6 cells/flask) into 25 cm^2 flasks with complete medium and then treated with GI_{50} value for each metal complexes for 1 – 4-24 h. Total RNA was extracted, quantified and 1 µg was reverse-transcribed. The complementary DNA (cDNA) was used as a template of qRT-PCR reactions. Data are expressed as fold change ± standard deviation in target gene expression in treated cells normalized to the internal control gene (GAPDH) and relative to the DMSO negative control. Fold change values >2 mean an up regulation of the target gene; fold change values < 0.5 mean a down regulation of the target gene. Basal expression was defined by a fold change ranging from 0.5 to 1.9

Chk1	$[Ni(tcitr)_2]$	$[Pt(tcitr)_2]$	$[Cu(tcitr)_2]$
**1h**	1.22±0.08	0.80±0.13	0.07±0.01
**4h**	22.10±1.32	0.42±0.14	0.05±0.01
**24h**	1.14±0.13	0.86±0.11	9.14±1.29

Table S8. Effects of [Ni(tcitr)$_2$], [Pt(tcitr)$_2$] and [Cu(tcitr)$_2$] on the expression of Cyclin A1. U937 cells were seeded (2×10^6 cells/flask) into 25 cm^2 flasks with complete medium and then treated with GI_{50} value for each metal complexes for 1 – 4-24 h. Total RNA was extracted, quantified and 1 µg was reverse-transcribed. The complementary DNA (cDNA) was used as a template of qRT-PCR reactions. Data are expressed as fold change ± standard deviation in target gene expression in treated cells normalized to the internal control gene (GAPDH) and relative to the DMSO negative control. Fold change values >2 mean an up regulation of the target gene; fold change values < 0.5 mean a down regulation of the target gene. Basal expression was defined by a fold change ranging from 0.5 to 1.9

Cyclin A1	[Ni(tcitr)$_2$]	[Pt(tcitr)$_2$]	[Cu(tcitr)$_2$]
**1h**	0.91±0.19	0.64±0.07	0.50±0.03
**4h**	1.72±0.54	0.40±0.03	0.23±0.01
**24h**	0.85±0.16	5.97±1.92	0.16±0.07

Table S9. Effects of [Ni(tcitr)$_2$], [Pt(tcitr)$_2$] and [Cu(tcitr)$_2$] on the expression of Cyclin B. U937 cells were seeded (2×10^6 cells/flask) into 25 cm^2 flasks with complete medium and then treated with GI_{50} value for each metal complexes for 1 – 4-24 h. Total RNA was extracted, quantified and 1 µg was reverse-transcribed. The complementary DNA (cDNA) was used as a template of qRT-PCR reactions. Data are expressed as fold change ± standard deviation in target gene expression in treated cells normalized to the internal control gene (GAPDH) and relative to the DMSO negative control. Fold change values >2 mean an up regulation of the target gene; fold change values < 0.5 mean a down regulation of the target gene. Basal expression was defined by a fold change ranging from 0.5 to 1.9

**Cyclin B**	**[Ni(tcitr)$_2$]**	**[Pt(tcitr)$_2$]**	**[Cu(tcitr)$_2$]**
**1h**	1.37 ± 0.24	0.40 ± 0.01	0.38 ± 0.06
**4h**	3.36 ± 0.78	0.24 ± 0.13	0.05 ± 0.00
**24h**	0.94 ± 0.06	0.71 ± 0.15	0.95 ± 0.02
